# Evaluation of the Association of Chewing Function and Oral Health-Related Quality of Life in a Population of Individuals Aged ≥ 45 Years and Residing in Communities in Switzerland: A Cross-Sectional Study

**DOI:** 10.3390/dj12060174

**Published:** 2024-06-06

**Authors:** Christian Tennert, Roberta Borg-Bartolo, Maria Prasinou, Maurus Kurt Jaeggi, Martin Schimmel, Andrea Roccuzzo, Guglielmo Campus

**Affiliations:** 1 Department of Restorative, Preventive and Pediatric Dentistry, School of Dental Medicine, University of Bern, 3012 Bern, Switzerland; roberta.borg-bartolo@unibe.ch (R.B.-B.); maria.prasinou@unibe.ch (M.P.); maurus.jaeggi@unibe.ch (M.K.J.); andrea.roccuzzo@unibe.ch (A.R.); guglielmo.campus@unibe.ch (G.C.); 2 Department of Reconstructive Dentistry and Geriodontology, School of Dental Medicine, University of Bern, 3012 Bern, Switzerland; martin.schimmel@unibe.ch; 3 Division of Gerodontology and Removable Prosthodontics, University Clinics of Dental Medicine, University of Geneva, 1205 Geneva, Switzerland; 4 Department of Periodontology, School of Dental Medicine, University of Bern, 3012 Bern, Switzerland

**Keywords:** oral health, mastication, epidemiology, clinical trial, tooth loss

## Abstract

Purpose: To analyse the association of masticatory performance and oral health-related quality of life in a representative population of individuals residing in communities in Switzerland aged ≥ 45 years. Materials and Methods: In total, 100 subjects completed two dedicated and validated questionnaires on their demographic data and the Geriatric Oral Health Assessment Index. A mixing ability test was performed for assessing masticatory performance. The qualitative analysis of the test was performed by categorizing the images into five categories, while the quantitative analysis was performed via a validated custom-made software. Results: Sixty-six samples could be analysed. Participants younger than 65 years of age showed significantly less frequent chewing deficiencies (17%) compared to those 65 years and older (50%, *p* < 0.01). However, retired participants had chewing deficiencies significantly more frequently (8%) compared to workers (51%, *p* < 0.01). A statistically significant positive association of having chewing deficiency was found between employment status (*p* < 0.01) and the presence of restorations (*p* = 0.04), while GOHAI did not show any statistically significant association. Overall, the enrolled subjects displayed moderate chewing function. Masticatory performance was positively associated with the number of present restorations. Conclusions: The enrolled subjects residing in communities in Switzerland aged ≥ 45 years displayed moderate chewing function. Their masticatory performance was positively associated with the number of present restorations but not associated with oral health related quality of life (GOHAI).

## 1. Introduction

The rise in life expectancy and the associated demographic changes are leading to a continuous increase in the elderly population in many countries. Despite the documented general improvement in oral health conditions, caries and periodontitis are still very prevalent and, if left untreated, lead to tooth loss [1,2]. A consequence of tooth loss is that chewing function deteriorates and dental prostheses may be required [1,3,4,5]. As a consequence of this condition, chewing function may be impaired and, with it, the food bolus cannot be formed appropriately. If the food bolus cannot be formed sufficiently due to inadequate chewing efficiency, food components, such as important nutrients are not sufficiently absorbed in the digestive system and this can result in malnutrition with regard to macro- and micronutrients [6,7,8]. This might affect general health in the long term. In a previous study, it was shown that a lack of chewing efficiency can no longer guarantee the supply of proteins in older people and can result in a loss of muscle mass, which can lead to a reduction in physical activity in the elderly [8]. In other studies, it was also found that dentures rated as inadequate tend to correlate with poorer chewing performance, which is particularly true for older people and can result in malnutrition or malnutrition [9,10]. Patients who still have their own teeth are at risk of developing further carious lesions, with root caries representing a significant problem, especially in older people. Periodontal diseases and the development of oral mucosal changes and even tumours are also favoured and often remain undetected [11].

The methods for objectively determining chewing efficiency can be categorised on the basis of comminution and the mixing and chewing performance tests of test foods. As early as 1950, Manly and Braley [12] used the sieve method to determine the chewing efficiency of previously chewed test food. However, the time required is relatively high, and the necessary availability of laboratory equipment as well as sieves and vibrators make implementation difficult [13]. For this reason, studies with optical analysis of the particles were later carried out for easier implementation. However, this also requires technical equipment and the purchase of image processing programmes [14]. Typical test foods are nuts, bread, fish, meat or vegetables. For the best possible comminution, however, the test food should have good breaking behaviour, as the food bolus must not clump together or disintegrate. Furthermore, it should be possible to grind it regardless of the type of denture or the number of occluding tooth pairs. Raw carrots, for example, fulfil these requirements; they initially have a largely homogeneous and comparable consistency, can be easily cut into a suitable shape, are available in fragments after chopping and do not clump together. They are also inexpensive and familiar to many patients as food, which means that a realistic chewing pattern can be achieved.

In addition to natural test foods, artificial test foods such as silicone cubes, hardened gelatine or fruit gums can be used. However, care should be taken to select the test material according to the patient’s performance in terms of masticatory muscles, chewing strength or dentition, as materials that are too hard may not be chewed [15]. Instead of comminution methods, mixing methods—with chewing gum or paraffin wax as test food—can be used to measure chewing efficiency, which show comparable results to comminution methods. One of the most used tests is a mixing test using a two-colour chewing gum, which makes it possible to assess the degree of colour mixture after 20 cycles of mastication and has been proved to be a good indicator for the ability of bolus formation. This test has been later improved by a digital analysis of the degree of colour mixture following specimen flattening to a thickness of 1 mm [16]. One of the most remarkable consequences of chewing impairment is the general deterioration of subjects’ well-being and quality of life. In this respect, dedicated scales have been developed to objectively assess the OHRQoL (i.e., OHIP-14 and GOHAI), including eating and chewing ability, even though these questionnaires do not include precise chewing ability measures [17,18]. Consequently, it is of paramount importance to investigate changes in oral health and chewing function. Hence, the aim of the present study was to analyse the association of masticatory function and the oral health-related quality of life of individuals aged ≥ 45 years residing in communities in Switzerland. It is expected that a decrease in masticatory performance is associated with lower oral health-related quality of life in the evaluated cohort.

## 2. Materials and Methods

### 2.1. Study Design

The study was designed a cross-sectional study. This paper reports a part of a larger research project. Data on caries and periodontal assessments of this cohort have been reported previously [19]. This study aimed to collect data on oral health, chewing function, nutrition, oral hygiene and oral health-related quality of life in adults/elderly citizens living in the Canton of Bern. The study protocol was approved by the Ethical Committee of the Canton of Bern (KEK), Switzerland (Nos. 2020-02760 and 2021-01947). The investigation was conducted according to the revised principles of the Helsinki Declaration (2013). Signed informed consent was obtained from each participant before participation in the study. A pilot study regarding clinical findings has been previously published where details on the clinical findings, such as dental status and periodontal assessment, can be retrieved [19].

### 2.2. Recruitment of the Subjects and Sample-Size Calculation

The citizens are registered at the Citizen Center of Bern. The contact information of the citizens of the Canton of Bern with an age of 45 years or older was provided by the Citizen Center of Bern. Information about this study was sent to the citizens of the Canton of Bern aged ≥ 45. Before being enrolled, each participant was required to provide written consent to participate in the study. Inclusion and exclusion criteria were as follows.

Inclusion criteria:

▪ Habitation/residence in the Canton of Bern;
▪ Written informed consent;
▪ Aged 45 years or older;
▪ Ability to understand and answer the questionnaire items.

Exclusion criteria:

▪ Known allergic reaction to oral hygiene products and/or medication and/or dental material previously used in the mouth or pharynx;
▪ Pathological changes of the oral mucosa, e.g., acute ulcerating gingivitis, acute herpetic gingivostomatitis, recurrent aphthous ulceration or systemic illnesses with oral manifestations;
▪ Antibiotic therapy within the past 3 weeks.

After written informed consent was obtained, questionnaires regarding oral hygiene, oral health, socio-economic level, life-style behaviours, and dietary assessment (items derived from nutritional assessment forms) were sent to the participants. An appointment was scheduled for the oral examination at the participant’s accommodation/residence by two trained and calibrated dentists. At this appointment, the questionnaires were collected and checked for completion. Any concerns about the questionnaires were discussed with the participant. Each participant was permitted to withdraw from the study at any stage of the observation. If a participant exhibited one or more exclusion criteria, their participation in this study was discontinued.

The sample size was assessed based on the frequency of mastication problems related to the number of teeth of citizens in the Canton of Bern aged ≥ 45 years, hypothesising a frequency of the outcome in the population category for this study of 70% (with a confidence interval of 5%) and a power of 90%. The number of subjects was set at 57. Subjects were selected taking proportional allocation into consideration: individuals from the ten different regions of the canton of Bern were recruited, and were selected by probability proportional to the size of the sample, according to the proposed STEP guidelines. 

Data reporting followed the STROBE guidelines.

### 2.3. Demographic Variables, GOHAI Assessment, and Clinical Examination

Participants completed two dedicated and validated questionnaires on their demographic data and the Geriatric Oral Health Assessment Index (GOHAI), developed by Atchison and Dolan [20,21]. This is a self-reported measure using a 12-item questionnaire evaluating oral health-related quality of life with scores in three domains: physical function, psycho-social function, and pain or discomfort [20]. All questionnaires were made available in German, French, and Italian. Clinical examination of the subjects was performed by two trained and calibrated dentists (AR and RBB). Caries score (ICDAS Codes) and restoration (filling) status; gingival bleeding (modified papilla bleeding index); inter-proximal plaque index; periodontal screening record (periodontal screening index); presence of prosthetic rehabilitation; and masticatory performance using a two-coloured chewing gum [19]. 

### 2.4. Masticatory Performance Assessment

Two experienced clinicians (AR or RBB) supervised the mixing ability test and collected the data. At the end of the clinical examination, subjects underwent a chewing gum test according to a methodology previously proposed and validated [16]. 

In brief, the test was performed using a two-colour mixing test (Hue-Check Gum©, University of Bern, Bern, Switzerland). Subjects were asked to introduce in their mouth two chewing gums with different colours (pink and blue) with the blue layer facing downwards and to normally perform 20 cycles of mastication. Thereafter, the gums were taken from the oral cavity of the subject, excess fluid was removed using a paper towel, and transferred into a transparent plastic bag. Subsequently, the specimens were flattened by one of the two investigators to a thickness of 1 mm using a resin template. 

**Figure 1:**
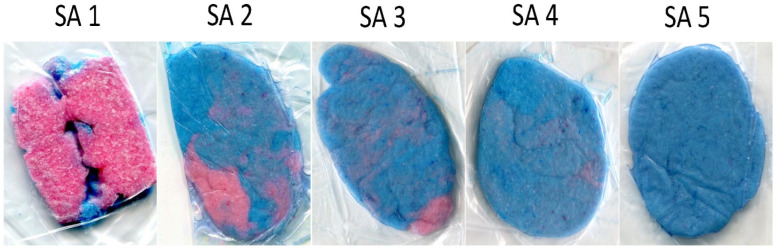
Examples of the different scores of subjective assessments (SA1–SA5).

The flattened chewing gum mix was placed on a white background and both sides were photographed (camera resolution: 8 megapixels). The qualitative analysis was performed categorizing the images into five categories of subjective assessment (SA) scores (Figure 1) [16]:

1. Score 1 (SA 1): chewing gum not mixed, impressions of cusps or folded once;
2. Score 2 (SA 2): large parts of chewing gum unmixed;
3. Score 3 (SA 3): bolus slightly mixed, but parts of unmixed original colour visible;
4. Score 4 (SA 4): bolus well mixed, but colour not uniform;
5. Score 5 (SA 5): bolus perfectly mixed with uniform colour.

One trained operator (F.G.) evaluated the images of the mixed and flattened chewing gums. For the purpose of this study, scores SA 1–3 were categorized as chewing deficiency (1) whereas score SA 4 and SA 5 were defined as no chewing deficiency (0).

### 2.5. Opto-Electronic Assessment: Variance of Hue (VoH)

A custom-made software (ViewGum ©, https://dhal.com, accessed on 15 January 2024) was used to analyse the colour mixture of the scans of the gums as described in detail previously [22]. The software converts the images of the specimens into the HSI (hue, saturation, intensity) colour space and calculates the homogeneity of the colour mixture as the variance of hue (VoH, range 0–1). In greater detail, the well-chewed specimens with a high degree of colour mixture present with a low VoH and vice versa. A quasi-logarithmic association of VoH and the number of chewing cycles, and masticatory performance has been previously reported [22]. All images of the mixed and flattened chewing gums, taken by mobile phone and scanner, were examined in this way by one trained operator (F.G.) with extensive experience in this procedure. 

### 2.6. Data Analysis

All data were collected in a Microsoft Excel^®^ (Microsoft^®^ Office 2016, Microsoft^®^ Corp, Redmond, WA, USA) spreadsheet and a researcher performed the quality check ensuring the accuracy of data collection. Descriptive statistics were calculated for all items to provide an overview of the results. When the response variable was a count, a square root transformation was performed among the groups to avoid the attenuating effect of unequal variability. The c^2^ test was used to test the independence of the qualitative variables. A predictive model for chewing deficiencies was performed using a logistic regression model via a forward stepwise regression procedure. The Cochrane–Armitage (Carmitage) trend test was performed to assess trends between the binary outcome variable (presence/absence of chewing deficiency) and questionnaire items and oral health status.

Statistical analysis was performed using Stata SE17^®^ (StataCorp LLC, College Station, TX, USA) with statistical significance set at *p* < 0.05.

## 3. Results 

A total of 100 subjects (response rate = 7.00%; males *n* = 63; mean age of 73 years, smokers *n* = 11) were included. Thirty-two subjects refused to undergo chewing performance testing and two chewing tests presented artefacts or failure of the scanning and analysis process. Consequently, 66 chewing tests could be analysed. The majority (*n* = 40) were males with a mean age of 70 years (SD 10.97) (range 45–91). Most of the participants were 65 years or older (82%), had a high educational status (61%), and were retired (75%) (Table 1). 

With respect to periodontal conditions, 46% of the subjects had PSI scores of 3–4. The mean DMFT score among the subjects was 13.35 (SD 5.38, range 1–28). With increasing age an increased trend of having less teeth (*p* = 0.03) and a decrease in the number of filled teeth (*p* = 0.00) was found. Regarding dentures, 81% of the subjects had fixed prostheses (bridges) (*p* = 0.02) and 22% had restorations on implants. Eleven percent of the subjects, all aged more than 75 years, had removable dentures. Analysing functional units (defined as a pair of opposing natural or prosthetic teeth, excluding third molars), most of the subjects had 11–14 occlusal functional units (84%), while 16% had 6–10 occlusal units.

Table 2 shows the distribution of the subjects with and without chewing deficiencies and their association with different variables. Chewing deficiencies were defined as scores 1–3, whereas scores 4 and 5 were defined as no chewing deficiency.

Participants younger than 65 years of age showed significantly less frequent chewing deficiencies (17%) compared to those 65 years and older (50%, *p* < 0.01). Gender and educational level did not show a significant association with chewing performance. Retired participants had chewing deficiencies significantly more frequently (8%) compared to workers (51%, *p* < 0.01). However, as employment status is most likely to be related to age, a test for confounding was carried out and the stratum-specific OR (OR 0.5, CI 0.04–5.99) compared to the crude OR (OR 0.08, CI 0.009–0.66). As the OR differ, age is considered to be a confounder.

The mean GOHAI among all subjects was 45 (SD 3.95) (range 30–60). Participants who reported GOHAI grades 1 and 2 on the GOHAI questionnaire were defined as low GOHAI, whereas grades 3–5 were defined as high GOHAI. A reported low GOHAI was significantly associated with having chewing deficiencies (88%), while participants reporting high GOHAI grades had chewing problems significantly less frequently (63%, *p* < 0.01).

With respect to dental conditions (number of functional units, defined as a pair of opposing natural or prosthetic teeth, excluding third molars), the majority of the subjects (*n* = 57, 86%) had more than 20 teeth present and were not wearing removable prosthesis (*n* = 60, 91%). None of the other investigated variables (i.e., number of functional units, wearing a prosthesis, having at least one crown, and no or at least one dental implant) showed a statistically significant association with chewing performance (*p* > 0.05).

The multinomial regression probit revealed a statistically significant positive association between employment status (*p* < 0.01) and the presence of restorations in terms of crowns (i.e., single crowns (*p* = 0.04)) with having a chewing deficiency, while GOHAI did not show any statistically significant association (Table 3).

## 4. Discussion

This study investigated the chewing performance of a representative cohort of Swiss citizens aged 45 years and older randomly selected in the Canton of Bern, Switzerland. The majority of the enrolled subjects displayed good to optimal chewing abilities (SA 4-SA 5) and their ability was associated with the number of present teeth and the presence of a removable prostheses. The applied colorimetric chewing test had been previously tested and its reliability validated [16,23]. Very recently, a study from Geneva (Switzerland) evaluated different chewing performance tests including the glucose extraction test (Jelly scan) and the colorimetric chewing test. The authors found colour-changing gum tests to be a sufficient screening tool for poor masticatory function. However, the sensitivity of this method is moderate, as a previous study found that, in cases where subjects show poor masticatory function, there is the risk of underdiagnosing masticatory function [24]. 

One aspect common to all the aforementioned studies is that they did not include oral health-related quality of life parameters. On the other hand, the present investigation is, to the best of the authors’ knowledge, the first report on oral health and masticatory function, including masticatory performance, to assess the OHRQoL in Swiss citizens aged ≥ 45 years in the Canton of Bern, Switzerland.

In previous studies, different questionnaires have been used to assess oral health-related quality of life (OHRQoL). For the elderly population, especially, a specific measure of OHRQoL was needed. The German Oral Health Impact Profile is a questionnaire from 14 to 53 items. This questionnaire could be challenging for many elderly people. The GOHAI aims to be a measure that is relatively compact so as to achieve a high response rate and the compliance of the elderly subjects taking part in the survey [25,26]. 

The reported mean GOHAI of 45 is lower than those reported in neighbouring countries [27,28]. Subjects with fair or poor oral health showed low GOHAI scores, a low sense of well-being regarding oral health, and an awareness of dental care requirements. As reported previously, the mean GOHAI score decreased with aging [26]. Mean GOHAI scores seem not to be influenced by gender, location of residence (rural or urban), or marital status. In contrast, low levels of education and a low income have been shown to negatively impact GOHAI [25]. As previously reported, subjects with a high GOHAI have shown a higher number of present teeth, lower carious scores, and fewer missing teeth. GOHAI is also affected by the presence of one or more removable prostheses, resulting in lower GOHAI scores than participants who did not use a denture. In the investigated cohort, chewing performance among the subjects was found to be of moderate quality. Categories SA 4 and SA 5 were found in 44% of the subjects, resulting in good (score 4) or perfectly (score 5) mixed chewing gums, while 56% of the subjects showed categories SA 1–3, which means a moderate-to-poor chewing performance. This might be speculated to be indirect evidence of the overall quality of the oral health care system in Switzerland. However, more than 50% of the evaluated cohort revealed deficient or even insufficient chewing performance. The main factors influencing chewing efficiency are the number of tooth pairs present and their contact with each other (occlusion). The lower the number of tooth pairs, the lower the masticatory efficiency. The posterior teeth determine a large part of the masticatory efficiency [4,29,30]. If the number of occluding tooth pairs is less than 8, masticatory efficiency is considered to be reduced [30]. Diseases such as periodontitis, caries, or tooth loosening can reduce chewing efficiency [31]. In addition to natural teeth, the presence and type of dentures also influence masticatory efficiency, whereby removable dentures, in particular, cannot replace the masticatory efficiency of a naturally fully dentate patient [4]. While implant-supported dentures still produce the highest masticatory efficiency, the mucosa under the supporting surface in combination with the lack of direct force transmission into the jawbone by teeth or implants limits the masticatory force of partial dentures. Patients with complete dentures therefore have the lowest masticatory efficiency, as they are completely supported by the mucosa [31,32].

In addition to the chewing test principles used in this study, other methods are known and used because the colour mixing of chewing gum is directly related to the material properties. At this point, rheological factors and the associated change in hardness with increasing chewing make it difficult to compare different mixing tests [33]. In another method, the glucose content or the colour change in the saliva is measured after a defined number of chewing cycles of fruit gums. Similarly, after chewing on a capsule containing dye beads, the change is evaluated using a spectrophotometer [33]. It should be borne in mind that chewing on a capsule is difficult to compare with a natural chewing process. The design and condition of dentures also have a significant influence on masticatory efficiency. This is reduced when the fit and retention of the retaining elements decrease. This process has a more negative influence than the loss of a pair of occluding teeth [32].

This study is not free from limitations: indeed, it has to be recalled that the overall response rate was very low (i.e., 7.00%), mainly due to technical issues in the acceptance of the reached subjects due to post COVID-19 social concerns and the fact that more than 30% of the samples could not be analysed. In addition, data gathered from self-reported patient questionnaires are always prone to collection and interpretation bias. Moreover, the categorization of the analysed cohort according to the subjects’ age (i.e., <65 years and ≥65 years) was based on the authors’ assumption that, in accordance with the WHO criteria, elderly people can be defined as such after 65 years of age. Consequently, the external validity of the obtained data to this Swiss population is questionable. The results of the colour mixing method by Schimmel et al. [23] used in this study show that a high standard deviation represents a higher proportion of unmixed chewing gum and, therefore, a lower chewing efficiency. The test does not show a direct instruction for action, but rather a continuous value for the degree of mixing. Another limitation of the gum mixing test is that these chewing gums soften during chewing and therefore become easier to chew. Mixing the colours of the two-coloured chewing gum is easily accomplished by subjects with good chewing capacity. From a certain degree of colour mixing, a saturation effect occurs, whereby fine differences between individuals cannot be precisely discriminated [34].

On the patient side, there is also the “paradox of ageing” [35]. This is clearly illustrated by the example of dentures, for which patients often have low expectations as they become older. This can lead to “underreporting”, meaning that problems with existing dentures are often recognised and treated too late or not at all [36].

## 5. Conclusions

The enrolled subjects aged ≥ 45 years and residing in communities in Switzerland displayed moderate chewing function. The masticatory performance was positively associated with the number of present crowns but not associated with oral health-related quality of life (GOHAI).

## Figures and Tables

**Table 1:** Demographic characteristics of the sample included.

Variables	Number of Subjects (%)
Age	
<65 years	12 (18.80)
≥65 years	54 (81.82)
Sex	
Male	40 (60.61)
Female	26 (39.39)
Educational status	
Low-medium grade	26 (39.22)
High grade	40 (60.78)
Working status	
Workers	13 (25.00)
Retired	39 (75.00)
No replies *n* = 14 (21.21)	

**Table 2:** Distribution of chewing deficiencies and association with questionnaire variables.

Chewing Deficiencies		
	Not Present *n* (%)	Present *n* (%)
<65 years of age	10 (83.33)	2 (16.67)
≥65 years of age	27 (50.00)	27 (50.00)
χ^2^_(1)_ = 4.43 *p* = 0.03 Carmitage trend z = 2.10 *p* = 0.03		
Male	23 (57.50)	17 (42.50)
Female	14 (53.85)	12 (46.15)
χ^2^_(1)_ = 0.09 *p* = 0.77 Carmitage trend z = 0.29 *p* = 0.77		
Low-medium educational level	9 (45.00)	11 (55.00)
High educational level	21 (67.74)	10 (32.26)
χ^2^_(1)_ = 2.60 *p* = 0.11 Carmitage trend z = −1.61 *p* = 0.11		
Workers	12 (92.31)	1 (7.69)
Retired	19 (48.72)	20 (51.28)
1-Fisher exact *p* < 0.01 Carmitage trend z = −2.77 *p* < 0.01		
Low GOHAI	1 (12.50)	7 (87.50)
High GOHAI	35 (62.50)	21 (37.50)
1-Fisher exact *p* = 0.01 Carmitage trend z = −2.67 *p* < 0.01		
≤20 functional units	4 (33.33)	8 (66.67)
21–24 functional units	12 (63.16)	7 (36.84)
>24 functional units	21 (60.00)	14 (40.00)
1-Fisher exact *p* = 0.24 Carmitage trend z = −1.33 *p* = 0.19		
No fixed prosthetic (bridges)	30 (60.00)	20 (40.00)
Fixed prosthetic (bridges)	7 (43.75)	9 (56.25)
χ^2^_(1)_ = 1.30 *p* = 0.25 Carmitage trend z = 1.14 *p* = 0.25		
No crowns	9 (47.37)	10 (52.63)
At least one crown	28 (59.57)	19 (40.43)
χ^2^_(1)_ = 0.82 *p* = 0.37 Carmitage trend z = −0.91 *p* = 0.37		
No implant	32 (59.26)	22 (40.74)
At least one implant	5 (41.67)	7 (58.33)
χ^2^_(1)_ = 1.23 *p* = 0.27 Carmitage trend z = 1.11 *p* = 0.27		

Pearson’s chi-square or Fisher’s exact test were applied if a cell had a value <5; the Cochrane–Armitage (Carmitage) trend test was applied between binary outcome variable (presence/absence of chewing deficiency) and questionnaire items and oral health status.

**Table 3:** Multinomial regression predictive model via a forward procedure. The presence of chewing deficiencies was the dependent variable.

Log Likelihood = −28.43	Observations = 62		χ^2^_(3)_ = 13.28	*p* < 0.01
Covariates	Coeff ± SD	*p*-Value	_95%_CI	
Working status	1.77 ± 0.58	<0.01	0.63–2.91	
GOHAI	−0.12 ± 0.11	0.27	−0.34–0.10	
Dental Crown	−0.91 ± 0.50	0.04	−1.58–0.07	

## Data Availability

Data is unavailable due to privacy and ethical restrictions.

## References

[1] Naka O., Anastassiadou V., Pissiotis A. (2014). Association between functional tooth units and chewing ability in older adults: A systematic review. Gerodontology.

[2] Zenthofer A., Ehret J., Zajac M., Kilian S., Rammelsberg P., Klotz A.L. (2020). The Effects of Dental Status and Chewing Efficiency on the Oral-Health-Related Quality of Life of Nursing-Home Residents. Clin. Interv. Aging.

[3] Barbe A.G., Javadian S., Rott T., Scharfenberg I., Deutscher H.C.D., Noack M.J., Derman S.H.M. (2020). Objective masticatory efficiency and subjective quality of masticatory function among patients with periodontal disease. J. Clin. Periodontol..

[4] Fontijn-Tekamp F.A., Slagter A.P., Van Der Bilt A., Van T.H.M.A., Witter D.J., Kalk W., Jansen J.A. (2000). Biting and chewing in overdentures, full dentures, and natural dentitions. J. Dent. Res..

[5] Kosaka T., Ono T., Kida M., Kikui M., Yamamoto M., Yasui S., Nokubi T., Maeda Y., Kokubo Y., Watanabe M. (2016). A multifactorial model of masticatory performance: The Suita study. J. Oral Rehabil..

[6] Brodeur J.M., Laurin D., Vallee R., Lachapelle D. (1993). Nutrient intake and gastrointestinal disorders related to masticatory performance in the edentulous elderly. J. Prosthet. Dent..

[7] Minakuchi S., Tsuga K., Ikebe K., Ueda T., Tamura F., Nagao K., Furuya J., Matsuo K., Yamamoto K., Kanazawa M. (2018). Oral hypofunction in the older population: Position paper of the Japanese Society of Gerodontology in 2016. Gerodontology.

[8] Remond D., Machebeuf M., Yven C., Buffiere C., Mioche L., Mosoni L., Patureau Mirand P. (2007). Postprandial whole-body protein metabolism after a meat meal is influenced by chewing efficiency in elderly subjects. Am. J. Clin. Nutr..

[9] Lee I.C., Yang Y.H., Ho P.S., Lee I.C. (2014). Chewing ability, nutritional status and quality of life. J. Oral Rehabil..

[10] Motokawa K., Mikami Y., Shirobe M., Edahiro A., Ohara Y., Iwasaki M., Watanabe Y., Kawai H., Kera T., Obuchi S. (2021). Relationship between Chewing Ability and Nutritional Status in Japanese Older Adults: A Cross-Sectional Study. Int. J. Environ. Res. Public Health.

[11] Wostmann B., Seelbach M., Seelbach P., Podhorsky A., Kolb G.F., Bretzel R.G., Rehmann P. (2017). Mini dental assessment: A simple screening test for non-dental staff. Clin. Oral Investig..

[12] Manly R.S., Braley L.C. (1950). Masticatory performance and efficiency. J. Dent. Res..

[13] Schmidt A., Schlenz M.A., Gabler C.S., Schlee S., Wostmann B. (2021). Development of a New Application-Based Chewing Efficiency Test (Mini Dental Assessment) and Its Evaluation by Nursing Staff in Geriatric Care: A Pilot Study. Int. J. Environ. Res. Public Health.

[14] Elgestad Stjernfeldt P., Sjogren P., Wardh I., Bostrom A.M. (2019). Systematic review of measurement properties of methods for objectively assessing masticatory performance. Clin. Exp. Dent. Res..

[15] Bates J.F., Stafford G.D., Harrison A. (1976). Masticatory function—A review of the literature. III. Masticatory performance and efficiency. J. Oral Rehabil..

[16] Schimmel M., Rachais E., Al-Haj Husain N., Muller F., Srinivasan M., Abou-Ayash S. (2022). Assessing masticatory performance with a colour-mixing ability test using smartphone camera images. J. Oral Rehabil..

[17] Inukai M., John M.T., Igarashi Y., Baba K. (2010). Association between perceived chewing ability and oral health-related quality of life in partially dentate patients. Health Qual. Life Outcomes.

[18] Klotz A.L., Hassel A.J., Schroder J., Rammelsberg P., Zenthofer A. (2017). Oral health-related quality of life and prosthetic status of nursing home residents with or without dementia. Clin. Interv. Aging.

[19] Roccuzzo A., Borg-Bartolo R., Schimmel M., Tennert C., Manton D.J., Campus G. (2023). Evaluation of the Oral Health Conditions and Oral Health-Related Quality of Life in a Community-Dwellers Population Aged ≥ 45 Years in the Canton of Bern: A Preliminary Pilot Study. Int. J. Environ. Res. Public. Health.

[20] Atchison K.A., Dolan T.A. (1990). Development of the Geriatric Oral Health Assessment Index. J. Dent. Educ..

[21] Swoboda J., Kiyak H.A., Persson R.E., Persson G.R., Yamaguchi D.K., MacEntee M.I., Wyatt C.C. (2006). Predictors of oral health quality of life in older adults. Spec. Care Dent..

[22] Buser R., Ziltener V., Samietz S., Fontolliet M., Nef T., Schimmel M. (2018). Validation of a purpose-built chewing gum and smartphone application to evaluate chewing efficiency. J. Oral Rehabil..

[23] Schimmel M., Christou P., Miyazaki H., Halazonetis D., Herrmann F.R., Muller F. (2015). A novel colourimetric technique to assess chewing function using two-coloured specimens: Validation and application. J. Dent..

[24] Imamura Y., Chebib N., Ohta M., Mojon P., Schulte-Eickhoff R.M., Schimmel M., Graf C., Sato Y., Muller F. (2023). Masticatory performance in oral function assessment: Alternative methods. J. Oral Rehabil..

[25] Hassel A.J., Rolko C., Koke U., Leisen J., Rammelsberg P. (2008). A German version of the GOHAI. Community Dent. Oral Epidemiol..

[26] Tubert-Jeannin S., Riordan P.J., Morel-Papernot A., Porcheray S., Saby-Collet S. (2003). Validation of an oral health quality of life index (GOHAI) in France. Community Dent. Oral Epidemiol..

[27] Klotz A.L., Tauber B., Schubert A.L., Hassel A.J., Schroder J., Wahl H.W., Rammelsberg P., Zenthofer A. (2018). Oral health-related quality of life as a predictor of subjective well-being among older adults-A decade-long longitudinal cohort study. Community Dent. Oral Epidemiol..

[28] Maitre I., Lourtioux F., Picouet P., Braud A. (2020). Oral health-related food selectivity among French independently living elders. J. Oral Rehabil..

[29] Kim H.E., Lee H. (2021). Factors affecting subjective and objective masticatory function in older adults: Importance of an integrated approach. J. Dent..

[30] Lu T.Y., Chen J.H., Du J.K., Lin Y.C., Ho P.S., Lee C.H., Hu C.Y., Huang H.L. (2020). Dysphagia and masticatory performance as a mediator of the xerostomia to quality of life relation in the older population. BMC Geriatr..

[31] Muller F., Nitschke I. (2005). Oral health, dental state and nutrition in older adults. Z. Gerontol. Geriatr..

[32] Klotz A.L., Ehret J., Zajac M., Schwindling F.S., Hassel A.J., Rammelsberg P., Zenthofer A. (2020). The effects of prosthetic status and dementia on the chewing efficiency of seniors in nursing homes. J. Oral Rehabil..

[33] Goncalves T., Schimmel M., van der Bilt A., Chen J., van der Glas H.W., Kohyama K., Hennequin M., Peyron M.A., Woda A., Leles C.R. (2021). Consensus on the terminologies and methodologies for masticatory assessment. J. Oral Rehabil..

[34] van der Bilt A. (2011). Assessment of mastication with implications for oral rehabilitation: A review. J. Oral Rehabil..

[35] Araujo L., Teixeira L., Ribeiro O., Paul C. (2018). Objective vs. Subjective Health in Very Advanced Ages: Looking for Discordance in Centenarians. Front. Med..

[36] Slagter A.P., Olthoff L.W., Bosman F., Steen W.H. (1992). Masticatory ability, denture quality, and oral conditions in edentulous subjects. J. Prosthet. Dent..

